# Effects of *Tetraselmis chuii* Microalgae Supplementation on Anthropometric, Hormonal and Hematological Parameters in Healthy Young Men: A Double-Blind Study

**DOI:** 10.3390/ijerph19106060

**Published:** 2022-05-16

**Authors:** Ángel García, Víctor Toro-Román, Jesús Siquier-Coll, Ignacio Bartolomé, Diego Muñoz, Marcos Maynar-Mariño

**Affiliations:** 1 School of Sport Sciences, University of Extremadura, Avenida de la Universidad s/n, 10003 Cáceres, Spain; angelpf100@gmail.com (Á.G.); ibartolomesa@upsa.es (I.B.); diegomun@unex.es (D.M.); mmaynar@unex.es (M.M.-M.); 2 SER Research Group, Center of Higher Education Alberta Giménez (Affiliated to Comillas Pontifical University), 07011 Palma de Mallorca, Spain; jesussiquier@cesag.org

**Keywords:** microalgae, anabolic, erythropoiesis, immune

## Abstract

The aim of this study was to evaluate the effects of *Tetraselmis chuii* (TC) microalgae supplementation for sixty days on hematological, anthropometric and hormonal parameters in healthy young men. Forty-six men divided into a placebo group (PG; *n* = 16; 20.77 ± 2.7 years; 72.14 ± 7.18 kg; 1.76 ± 0.07 m), a group supplemented with 25 mg/day of TC (SG 25; *n* = 15; 20.40 ± 1.40 years; 71.28 ± 8.26 kg; 1.76 ± 0.05 m) and another group supplemented with 200 mg/day of TC (SG 200; *n* = 15; 20.83 ± 2.45 years; 72.30 ± 11.13 kg; 1.77 ± 0.08 m) participated in this double-blind study. PG ingested 200 mg/day of lactose powder. Participants underwent 4 assessments (baseline, month 1, month 2 and desadaptation) separated in time by an interval of thirty days. At SG 25 and SG 200, significant increases in percent muscle mass, erythropoietin, insulin-like growth factor 1, free testosterone, leukocytes, neutrophils and lymphocytes were observed (*p* < 0.05). Decreases in the levels of percent fat mass, platelets, hematocrit and mean corpuscular hemoglobin also occurred in these groups (*p* < 0.05). TC supplementation induced favorable changes on anthropometric, hematological and hormonal levels. In view of the data, it seems that the most effective dose was 25 mg/day of TC.

## 1. Introduction

Microalgae are unicellular prokaryotic or eukaryotic microorganisms that reside in riverine and marine systems [1,2]. The nutritional potential of microalgae has been known to humans for thousands of years [3,4]. It should be noted that microalgae have a wide biodiversity, and it is estimated that there could be more than 200,000 different species on our planet [5]. However, the most researched species have been: *Chlorella*, *Spirulina*, *Haematococcus*, *Dunaniella* and *Scenedesmus* [1].

It is now widely recognized that microalgae synthesize a large variety of bioactive molecules, such as peptides, lipids, and other pigments, with different effects on human physiology [6]. Hence, both in the health and sports fields, there has been a growing interest in the knowledge of the biochemical effects that could be triggered by the consumption of these microorganisms [7,8]. In this respect, microalgae bioactive peptides act through biosignaling mechanisms similar to the signaling mechanisms of hormones [9]. These peptides are released into the bloodstream after digestive processes, generating different antihypertensive, antiobesity, antiatrophic, anti-inflammatory, anticarcinogenic, antidiabetic and immunomodulatory effects [9,10,11]. Moreover, the high content of polysaccharides, polyunsaturated fatty acids (PUFA), pigments and other lipid compounds confer on microalgae the ability to generate a wide range of interesting physiological effects, such as the stimulation of hematopoiesis, antioxidant systems, and some neuroendocrine axes, which remain the subject of continuous research [12,13,14].

The genus *Tetraselmis chuii* (TC) is a type of unicellular marine green microalgae belonging to the class of Prasinophyceae [15]. It was isolated decades ago, being widely used in the aquaculture industry [16]. Nonetheless, the possible effects of *Tetraselmis chuii* (TC) on the human organism remain unknown, and few authors have been interested in their research. The scarce scientific literature does not support the notable composition of TC in terms of essential amino acids, PUFA, vitamins and minerals [17,18]. Recently, it has been shown that this microalga possesses significant concentrations of antioxidant enzymes that could considerably decrease the rates of oxidative stress both in vivo in humans and in vitro in myoblasts [19] and in vitro in human skeletal muscle myoblasts [20]. Therefore, the promising results obtained in a preliminary study by Toro et al. (2020), where the effects of TC supplementation in athletes were evaluated, prompted the design of the present investigation with the aim of overcoming some of its limitations and complementing its findings [7]. Among them, to analyze the effects of different doses of TC to discover the effective dose.

The need for this research is substantiated by the interesting composition of TC and the limited number of publications that report on the effects of TC supplementation in humans. Hence, the main objective of this work was to evaluate the effects of sixty-day supplementation with TC on hematological, anthropometric, biochemical, and hormonal parameters in healthy young men.

## 2. Materials and Methods

### 2.1. Subjects

Forty-six healthy male students participated in the study. The groups were divided into a placebo group (PG; *n* = 16; 20.77 ± 2.7 years; 72.14 ± 7.18 kg; 1.76 ± 0.07 m), a group supplemented with 25 mg TC (*n* = 15; SG 25; 20.40 ± 1.40 years; 71.28 ± 8.26 kg; 1.76 ± 0.05 m) and a group supplemented with 200 mg TC (*n* = 15; SG 200; 20.83 ± 2.45 years; 72.30 ± 11.13 kg; 1.77 ± 0.08 m). Groups were randomly composed via a web page (https://www.randomizer.org/, accessed on 6 March 2017).

Participants were informed about the purpose of the study and voluntarily signed an informed consent before enrollment and start of the experimental phases. This research was conducted under the ethical guidelines of the Declaration of Helsinki, updated at the World Medical Assembly in Fortaleza (Brazil) in 2013 for research involving human subjects (registration code: 99/2016). Commitment to confidentiality was maintained with participants by assigning alpha-numeric codes in order to collect samples and treat data anonymously.

To participate in the study, the following inclusion criteria were followed: (i) no hematological alterations; (ii) last blood count within the reference ranges; (iii) no anemia of any type; (iv) no other supplementation during the study; (v) no changes in physical activity and nutritional habits during the experimental period; (vi) being a man. In addition, subjects had to meet all the criteria at least three months before the beginning of the study.

Participants completed a physical activity questionnaire [21] at the beginning of the study in order to monitor physical activity levels, and no significant differences (*p* < 0.05) were found among the different study groups. Similarly, they underwent a medical examination to evaluate possible contraindications without finding any complications.

### 2.2. Study Design

The present investigation followed all the methodological guidelines proposed by Toro et al. (2020) [7].

The research was carried out using a double-blind design. Participants belonging to SG 25 and SG 200 ingested a daily capsule of 25 mg and 200 mg, respectively, of TC powder (TetraSOD^®^, El Puerto de Santa Maria. Andalusia, Spain). Table 1 displays the data for the composition of TetraSOD^®^.

In contrast, PG ingested a 200 mg placebo tablet containing lactose powder. The nutritional value of the daily dose of placebo was 20.41 kcal, 0.4 g of water, 4.8 g of carbohydrates, 0.1 g of protein and 0.09 g of lipids. The supplementation protocol lasted 60 days. To avoid possible interpretations in both researchers and participants, both capsules had exactly the same design and characteristics. Participants were informed that the daily dose of TC was to be taken in the morning combined with breakfast. 

Four different evaluations were performed: the previous day before starting supplementation (baseline), after 30 days of supplementation (month 1), following 60 days of supplementation (month 2) and a final evaluation after 30 days without ingesting the supplement (desadaptation).

### 2.3. Anthropometry

Anthropometric characteristics were always evaluated by the same researcher, specialized in kinanthropometry techniques, at the same time and under fasting conditions following the guidelines of the International Group of Kinanthropometry [22]. The following were used for this purpose: a Seca scale© 769 (Seca, Hamburg, Germany), with an accuracy of ±100 g; a wall measuring rod with an accuracy of ±1 mm (Seca, Hamburg, Germany); a Seca brand tape© 201 (Seca, Hamburg, Germany), accurate to ±1 mm; a Holtain plicometer© 610ND (Holtain, Crymych, UK), accurate to ±0.2 mm; and a Holtain pachymeter© 604 (Holtain, Crymych, UK), accurate to ±1 mm. The anthropometric measurements evaluated were: height, weight, skinfolds (abdominal, suprailiac, tricipital, thigh and leg), bone diameters (bi-styloid, humeral bi-epicondylar and femoral bi-epicondylar) and relaxed arm and leg muscle perimeters. The percentages of muscle mass and fat mass were obtained using the equations provided by the International Group of Kinanthropometry [22].

### 2.4. Nutritional Assessment

To ensure that participants’ diets remained unchanged throughout the study, participants completed a nutritional log the first and last week of the supplementation period. The log collected the participants’ diets for 4 days of the week, 3 of which were pre-assigned and 1 weekend day. The study participants had to indicate the frequency, type, and quantity (in grams) of each food consumed on those days. Subsequently, using food composition tables, the nutritional composition of the diets of all individuals was evaluated [23].

### 2.5. Blood Collection and Determination of Hematological Parameters

A 10 mL sample of blood was drawn from the antecubital vein under fasting conditions at 9:00 a.m. To avoid possible biochemical fluctuations due to circadian rhythms, the time of withdrawal was kept constant throughout the procedure. The sample was collected in a polypropylene tube. To determine hematological values, a 200 µL sample was taken from each blood tube and then placed in the coulter for subsequent biochemical analysis (Coulter Electronics LTD. model 6706319; Northwell Drive, Luton, UK).

### 2.6. Hormone Determination

Hormone determination was performed by ELISA (enzyme-linked immunosorbent assay) using an ER-500 model (Sinnowa, Germany) and applying commercial tests for: erythropoietin (EPO), cortisol (C), testosterone (TES), dehydroepiandrosterone (DHEA), insulin-like growth factor (IGF-1) and growth hormone (GH). Prior to each determination, a calibration process was used following the manufacturer’s recommendations. All inter- and intra-assay coefficients of variation were less than 10% for all biochemical analyses.

### 2.7. Statistical Analysis

Statistical analyses were performed with SPSS 20.0 for Windows (SPSS Inc., Chicago, IL, USA). The normality of the distribution of the variables was analyzed using the Shapiro–Wilk test and the homogeneity of variances with the Levene test. To establish intra-group differences, the Wilcoxon test was applied for related samples in nonparametric variables. To determine the statistical magnitude of the differences between the study groups, the Mann–Whitney U test was used for nonparametric variables. A *p* < 0.05 was considered statistically significant. The results were expressed as mean ± standard deviation. 

## 3. Results

The results obtained in each of the four evaluations carried out throughout the study are shown below. Table 2 presents the information collected from the nutritional record. There were no significant differences (*p* < 0.05) for any of the variables measured during the investigation.

Table 3 shows the values corresponding to the anthropometric evaluation of the three groups during the experimental period. A significant increase in weight (*p* < 0.05) was only observed during the last two evaluations compared to the initial values and at month 1 in PG. In SG 25 and SG 200 during month 2, increases in muscle percentage (*p* < 0.05) and significant decreases in fat percentage (*p* < 0.05) were observed compared to the initial evaluation. In addition, SG 200 significantly raised Ʃ6 skinfold values (*p* < 0.05) after cessation of supplementation compared to month 2 levels. Finally, in SG 25 a significant decrease in Ʃ6 skinfold (*p* < 0.05) was observed during month 2 with respect to baseline values.

Table 4 shows the data on the evolution of hormone levels in the three groups during the course of the study. Significant increases in EPO (*p* < 0.05) were observed in SG 25 and SG 200 after two months of supplementation compared to baseline levels. Likewise, a decrease in the values of this hormone was observed in SG 25 after the desadaptation period with respect to month 2 (*p* < 0.05). There were significant increases (*p* < 0.01) in IGF-1 values during month 2 with respect to initial levels in these two groups. Furthermore, in SG 25 during month 2 and desadaptation, increases in TES were obtained (*p* < 0.05) compared to baseline concentrations. With reference to SG 200, a significant decrease in GH levels (*p* < 0.05) was observed after the cessation of supplementation with respect to the values of month 1. In PG during desadaptation, increases in C concentrations (*p* < 0.05) were detected with respect to baseline values, in DHEA (*p* < 0.05) compared to those obtained in month 2, and in TES (*p* < 0.05) in relation to the concentrations of month 1. Moreover, among PG participants, a significant drop in GH levels (*p* < 0.05) was found after desadaptation with respect to both baseline values and the corresponding values at month 2. Finally, significant differences (*p* < 0.05) were obtained between SG 200 and PG in DHEA levels during month 2 as well as in TES concentrations during month 1.

Table 5 shows the blood values of white blood cells and platelets in the three groups during all the evaluations. It can be observed that PG significantly increased the concentration of leukocytes (*p* < 0.05) and neutrophils (*p* < 0.05) during month 2 and desadaptation period with respect to baseline levels. In SG 25, there were several significant increases (*p* < 0.05) in leukocyte concentrations (*p* < 0.05) during month 1 and lymphocyte values (*p* < 0.05) during month 2 compared to baseline levels. Likewise, in SG 25, after desadaptation, there were elevations in neutrophil levels (*p* < 0.05) with respect to baseline concentrations and month 1 as well as decreases (*p* < 0.05) in lymphocyte values in relation to the previous two months. Besides, there was a significant fall (*p* < 0.05) in the concentration of eosinophils during month 2 in comparison to the initial levels in this group. In SG 200, only significant increases (*p* < 0.05) in lymphocyte concentrations were observed after three months of the study compared to the initial evaluation. Finally, there were significant differences (*p* < 0.05) in monocyte and basophil levels during month 1 between SG 25 and PG, as well as in lymphocyte concentrations during month 2 (*p* < 0.05) between SG 25 and SG 200. Regarding platelet values, in PG there were only drops (*p* < 0.05) in months 1 and 2 with respect to baseline values. Significant decreases in platelet count (*p* < 0.05) and plateletcrit (*p* < 0.05) were observed in SG 25 at month 1 compared to baseline values. In addition, a significant drop (*p* < 0.01) in platelet levels at month 2 and at mismatch with respect to baseline concentrations was found in this group. Likewise, in SG 25 after desadaptation, significant decreases were observed in both MPV levels (*p* < 0.05) compared to month 1, and plateletcrit (*p* < 0.05) with respect to baseline values. Additionally, significant differences were found between SG 25 and PG in baseline platelet (*p* < 0.05) and plateletcrit (*p* < 0.05) levels, with such differences (*p* < 0.05) also being recorded in baseline plateletcrit between SG 25 and SG 200.

Table 6 includes the results obtained in the three groups in relation to the red series during the investigation. In SG 25 and SG 200, there were significant decreases in hematocrit (*p* < 0.05) after desadaptation with respect to the first two months, such as in HCM levels (*p* < 0.05) after the last evaluation, compared to the initial levels. Subsequently, in SG 25 and SG 200, there was an elevation in HCM (*p* < 0.05) during the desadaptation in comparison to months 1 and 2. Moreover, there was a significant rise in MCV values (*p* < 0.05) after the first two months with respect to baseline levels in PG. Afterward, there was a significant decrease (*p* < 0.05) in this variable compared to month 2 during desadaptation. Furthermore, a significant drop in HCM levels (*p* < 0.05) was detected in the GP after month 1 with respect to baseline values. 

**Figure 1:**
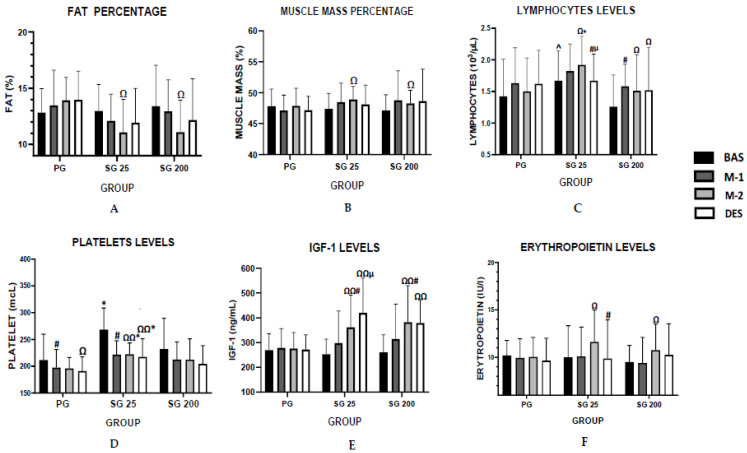
Key values: (**A**) fat percentage in the different evaluations; (**B**) muscle mass percentage in the evaluations; (**C**) lymphocytes levels in the different evaluations; (**D**) platelet levels evolution during the experimental period; (**E**) IGF-1 levels in the different evaluations; (**F**) erythropoietin levels in the different evaluations; SG 25: 25 mg group; SG 200: 200 mg group; BAS: baseline; M-1: month 1; M-2: month 2; DES: desadaptation; ^#^ *p* < 0.05 differences with respect to previous evaluation (paired samples Wilcoxon test); ^µ^ *p* < 0.05 differences desadaptation vs. month 1 (paired samples Wilcoxon test); ^Ω^ *p* < 0.05 desadaptation or month 2 vs. baseline (paired samples Wilcoxon test); ^Ω Ω^ *p* < 0.01 mismatch or month 2 vs. baseline (paired samples Wilcoxon test); * *p* < 0.05 differences placebo vs. SG 25 or SG 200 (Mann–Whitney U test; ^^^ *p* < 0.05 differences SG 25 vs. SG 200 (Mann–Whitney U test).

Figure 1 illustrates the key results obtained during the study. 

## 4. Discussion

The purpose of this research was to evaluate the effects of TC supplementation on different hematological, hormonal, and anthropometric parameters in young men students. Currently, there is little scientific literature assessing the effect of TC intake on human physiology. In this respect, this investigation has used as a reference the study carried out by Toro et al. (2020) where the effects of TC intake in athletes were evaluated, observing different hematological and ergo-spirometric changes [7]. Thus, we considered it interesting to evaluate the neuroendocrine response generated by the intake of TC and to elucidate possible mechanisms of action underlying these effects. Doses of 25 and 200 mg/d were chosen because the first amount was used in the abovementioned study. The second amount was because the highest dose studied is 250 mg/d, according to the Scientific Committee of the Spanish Agency for Food Safety and Nutrition [24].

In the supplemented groups, a multitude of endocrinological changes were observed. One of the most interesting endocrine phenomena revealed in this investigation was the significant increases in EPO concentrations (*p* < 0.05) only in the supplemented groups. The increase in EPO concentrations in the present study is in agreement with the findings evidenced by Toro et al. (2020), where a highly significant (*p* < 0.01) increase in hemoglobin levels was observed [7]. Previous research in animals where *Spirulina platensis* was administered demonstrated improvements in the red series, and it was hypothesized that these effects could be related to an increase in EPO [25]. In this line of thought, Hayashi et al. (2006) showed that certain pigments present in cyanobacteria such as C-phycocyanin can mimic the effects of EPO, increasing the proliferation of hematopoietic stem cells in the bone marrow of mice [26,27]. 

The mechanisms through which TC could increase endogenous EPO synthesis are currently unknown. In this regard, it should be considered that microalgae are rich in chlorides [28]. Specifically, cobalt chloride constitutes a well-known hypoxia agonist that stabilizes HIF-1 by promoting EPO synthesis [29,30]. Furthermore, the scientific literature has reported the existence of bioactive peptides in microalgae with a wide variety of effects [31]. Currently, no hematopoietic biopeptides have been reported as being present in TC, which could be of interest for future research in this field. Finally, the possible stimulation of the immune system due to the recognition of the polysaccharides present in TC could induce a detectable increase in EPO by the activation of certain macrophage strains that express the coding gene for the synthesis of this hormone [32,33,34].

Regarding TES, SG 25 showed an increase (*p* < 0.05) with respect to basal concentrations although it was not significant in SG 200. Concerning PG, TES values decreased (*p* < 0.05) except in the final evaluation when initial concentrations were reestablished probably due to some change in the lifestyle of the participants during the research. In relation to the results obtained, Farag et al. (2016) assessed the effects of *Spirulina platensis* supplementation on spermatogenesis and steroidogenesis in rats intoxicated with cadmium [35]. They concluded that *Spirulina platensis* supplementation increased the gene expression of the enzymes responsible for testicular steroidogenesis, thus reducing the harmful effects of cadmium. Other authors such as Sikiru et al. (2019) have evaluated the effects of *Chlorella vulgaris* ingestion on oxidative stress in rabbits [36]. They observed a reduction of lipid peroxidation in the supplemented group, protecting the function of Leydig cells. Thus, it is likely that the high antioxidant potential of microalgae may be related to an optimization of steroidogenesis [37,38].

In this regard, TC presents high activity of the enzyme superoxide dismutase (>30,000 U/g), as well as a rich composition in carotenoids and polyphenols, showing great antioxidant capacity [17,20,39]. Consequently, it is reasonable to assume that the recorded increases in TES in the supplemented groups are due to a cytoprotective effect of TC exerted on Leydig cells, optimizing testicular antioxidant mechanisms and, therefore, the function of steroidogenic enzymes.

With respect to C levels, they remained stable throughout the experimental period in both SG 25 and SG 200. Exceptionally, PG C concentrations increased (*p* < 0.05) after desadaptation compared to baseline levels and those at month 1. To interpret these results accurately, it is important to note that the participants performed the last two evaluations in very demanding academic periods. In this context, basal elevations of C have been widely identified in the literature as an adaptive mechanism to psycho-physiological stress [40]. Consequently, and considering the academic context of the assessments, this could be an explanation for the results obtained in PG. Lee et al. (2019) verified in pigs that supplementation with microalgae decreased C levels compared to supplementation with fish oil [41]. They concluded that it could be due to the content and type of omega-3 polyunsaturated fatty acid profile (n-3 PUFA) present in microalgae. This is in agreement with the findings of Robertson et al. (2017), who tested the effects of n-3 PUFA supplementation in female mice on corticosterone secretion, recording decreases in its values [42]. TC is rich in long-chain polyunsaturated fatty acids (PUFA), especially n-3 PUFA, eicosapentaenoic acid (EPA) and docosahexanoic acid (DHA) [17]. It has been shown that these fatty acids could decrease the catalytic activity of adrenal steroidogenic enzymes involved in the synthesis of C such as CYP 21 [43]. This would explain the greater degree of control of C concentrations obtained by the groups supplemented with TC in the face of possible academic stress, which did not occur in PG.

Concerning DHEA, concentrations of this hormone increased during desadaptation in SG 25 (*p* < 0.05) compared to basal values and in PG (*p* < 0.05) with respect to month 2. Several investigations have evaluated the effects of microalgae ingestion on DHEA. Chiu et al. (2021), in a double-blind randomized clinical trial, observed increases in DHEA related to the intake of a *Chlorella pyrenoidosa* extract beverage in 44 healthy participants [44]. To the best of our knowledge, this is the only study evaluating the possible effects of a microalga on this hormone in a human organism. In the research, no apparent effects of TC consumption on DHEA were apparent. Therefore, more research is needed to clarify these relationships.

Regarding GH, decreases (*p* < 0.05) were observed in both PG and SG 200 during de-adaptation with respect to initial values. The results obtained in PG could be based on the parallel increase in their C values (*p* < 0.05). In this line of thought, it is known that chronic increases in glucocorticoid levels suppress pituitary GH secretion [45]. On the other hand, in the supplemented groups it is interesting to note how GH levels decreased after desadaptation with respect to baseline values. It has been described that these changes could be associated with a negative impact on the pituitary, caused by the strong increase of IGF-1 concentrations in the groups supplemented with TC [46].

In relation to IGF-1, significant increases were observed in the supplemented groups (*p* < 0.01). However, in PG, IGF-1 concentrations remained unchanged. Currently, there is no solid evidence linking microalgae supplementation and the elevation of IGF-1 concentrations. Some studies such as that by Cho et al. (2020) observed highly significant increases in IGF-1 (*p* < 0.001) and IGFBP-3 levels after 7 weeks in rats supplemented with *Spirulina platensis* [47]. Consistent with these results, Fournier et al. (2016) evidenced increases in IGF-1 concentrations after 9 weeks of Spirulina supplementation in rats subjected to protein undernutrition [48]. 

Androgens could induce different stimulatory effects on the GH-IGF-1 axis [49,50]. In this regard, there is strong evidence that TES increases both GH secretion and its response in hepatic and muscle tissue [51]. In addition, TES has been shown to increase hepatic and muscular IGF-1 production independently of GH [49,52]. Based on this, the obtained increases in IGF-1 could be due to the increases in TES recorded in the groups supplemented with TC. 

Concerning hemogram parameters, platelet levels and volumes decreased in both PG (*p* < 0.05) and SG 25 (*p* < 0.01) in comparison to baseline values. However, decreases in platelet numbers in SG 25 were greater than in PG. Furthermore, only in SG 25 were significant decreases in MPV (*p* < 0.05) identified after supplement consumption. Villar et al. (1997) reported potent antiplatelet effects on human platelet extracts using the microalgae *Dunaliella tertiolecta* [53]. These authors concluded that this microalga had inhibitory effects on thrombin, arachidonic acid and iomycin, key elements for platelet aggregation [54,55]. Subsequently, Koukuraki et al. (2020) evaluated the antithrombotic properties of an extract of *Spirulina platensis* in rabbit platelets [56]. They observed that Spirulina showed potent thrombin inhibitory activity.

PUFAs are the components of TC that could have the strongest relationships with thrombopoiesis and platelet activity [57]. Marine n-3 PUFAs have been associated with reduced platelet counts, decreased platelet reactivity, longer bleeding times, and a lower ratio of pro-aggregating thromboxanes to anti-aggregating prostacyclins [58,59]. Similarly, compounds such as carotenoids and polysaccharides present in TC have been reported to exhibit antiplatelet properties [60,61]. These findings would imply that TC supplementation could contribute to the prevention of atherothrombotic pathology. Interestingly, the effect was not dose-dependent, as more discrete decreases in platelet count were recorded in SG 200 without reaching statistical significance.

The white series also underwent interesting changes. First, it should be noted that PG alone showed increases in neutrophil levels (*p* < 0.05) between desadaptation and month 2. As a consequence, this group demonstrated increased leukocyte counts (*p* < 0.05), following the same pattern as neutrophil values. On the other hand, in SG 25, increases in leukocyte (*p* < 0.05), neutrophil (*p* < 0.05), basophil (*p* < 0.05) and lymphocyte (*p* < 0.05) concentrations were observed. Curiously, increases in lymphocyte values were observed in SG 200 only (*p* < 0.05). Simpore et al. (2007) investigated the effects of spirulina on immune functions in children infected with human immunodeficiency virus (HIV) [62]. They concluded that supplementation with 10 mg per day of spirulina for one year improved the immune status of the patients, associating these results with an increase in CD4 T lymphocyte levels. Recently, Cai et al. (2022) identified the presence of an acidic polysaccharide in the composition of *Spirulina platensis*, named SP90-1 [63]. They observed in vitro in RAW264.7 cells that this substance promoted the secretion of nitric oxide, IL-1β, and TNFα. This could explain part of the immunomodulatory activity of *Spirulina platensis*.

It has been verified that the walls of the genus *Tetraselmis* sp. contain acidic and sulfated polysaccharides [64,65]. Several studies have shown that sulfation of the polysaccharides can enhance detection by TLR4 receptors of macrophages [65,66,67]. Upon recognition of the polysaccharides, TLR4 would increase the production of bone marrow-stimulating cytokines and chemokines [66,67]. This would increase the proliferation of the various immune cells, as well as their extravasation into the bloodstream [67].

In addition to these results, a modification of body composition was also observed in the supplemented groups with respect to that corresponding to PG. The percentage of muscle mass increased (*p* < 0.05) in SG 25 and SG 200 after supplementation with TC. Additionally, the percentage of fat mass decreased significantly (*p* < 0.05) in both groups. However, no relevant differences were found in PG. In the interpretation of these results, it should be considered that there were no variations in the diet of the participants throughout the study. Therefore, the anthropometric effects observed could be due to the supplementation with TC. 

These results seem to be in line with the hormonal changes described above. The increases in TES and IGF-1 concentrations in the supplemented groups could explain the recorded increases in lean mass at SG 25 and SG 200 [68,69]. Furthermore, the possible increase in GH sensitivity in the supplemented groups could influence both the decrease in fat percentage and the increase in muscle mass in these participants [70]. As mentioned above, the presence of FGF-21 in the histological compartments creates resistance to GH; this factor is also expressed to a considerable extent in adipose and muscle tissue. 

Moreover, it has been evidenced that microalgae could possess an anti-obesity effect through inhibition of pre-adipocyte differentiation and increased expression of lipolytic genes in adipose tissue [1]. Additionally, preclinical animal studies have shown that various components of microalgae could increase the activity of brown adipose tissue, increasing the basal metabolic rate and favoring lipolytic processes [71]. Finally, the existence of several bioactive components and peptides in microalgae with anti-atrophic and lipolytic functions has been reported [1,72]. Although the biochemical mechanisms on which TC could act in this context are uncertain, it is plausible that it is due to a combination of all the above effects.

This research has several limitations, and it is important to consider the scarcity of authors who have previously studied the effects of TC in humans. First, the sample of this research was small and did not include women. Second, the participants’ intake of important micronutrients, such as iron or b vitamins, was not analyzed. Third, neither iron nor reticulocyte levels of individuals were assessed, in order to analyze erythropoiesis more effectively. Fourth, although the participants confirmed at all times that they had not changed their lifestyle, it is noteworthy that only two objective nutritional controls were performed, at the beginning and at the end of the study. Therefore, there is a risk that the participants could have changed their eating habits during part of the experimental period. Fifth, platelet function could not be assessed to accurately verify the effects of TC on platelet aggregation. Sixth, the use of dual-energy X-ray absorptiometry (DEXA) could have provided more accurate data regarding changes in body composition. Seventh, oxidative stress was not assessed in the present work, which could contribute to explanations of the possible effects of TC on steroidogenesis. Eighth, only two different doses of CT were administered for this study. Finally, the short duration of the present study makes it difficult to objectively and accurately assess the long-term effects of CT supplementation.

## 5. Conclusions

The results obtained in the present work indicate that daily supplementation with 25 mg of TC (TetraSOD^®^) for sixty days could optimize hematological, hormonal, biochemical and anthropometric parameters in healthy male students. No additional improvements were observed with the intake of 200 mg of TC. The data obtained provide a physiological approach that could determine the potential use of this microalgae in both the sports and health fields although most of the endocrine-metabolic mechanisms underlying the effects of TC remain unknown at present. Future research is needed to clarify the pathways of action of TC in the human body.

## Figures and Tables

**Table 1:** Composition of TetraSOD^®^ from Fitoplacton marino S.L.

Component	Quantity	Component	Quantity
Protein(mg/pill)	75.2 ± 2.51	Manganese (mg/g)	5.06 ± 0.09
Carbohydrates (mg/pill	63.2 ± 2.67	Iodine (mg/kg)	5.03 ± 5.78
Lipids (mg/pill)	13.4 ± 1.04	Calcium (mg/g)	33.8 ± 0.26
Aspartic acid (mg/pill)	1.39 ± 0.06	Phosphorus (mg/g)	6.27 ± 1.87
Glutamic acid (mg/pill)	1.75 ± 0.08	Magnesium (mg/g)	5.06 ± 0.09
Saturated fatty acids (mg/pill)	4.06 ± 0.41	Sodium (mg/g)	14.33 ± 4.16
Monounsaturated fatty acids (mg/pill)	7.05 ± 0.86	Chloride (mg/g)	17.77 ± 0.25
Polyunsaturated fatty acids (mg/pill)	6.26 ± 0.77	Copper (mg/g)	0.006 ± 0.00
Leucine (mg/pill)	1.15 ± 0.08	Iron (mg/g)	2.01 ± 0.01
Arginine (mg/pill)	1.00 ± 0.06	Potassium (mg/g)	10.40 ± 0.56

**Table 2:** Nutritional assessment during the study.

		PG (*n* = 16)	SG 25 (*n =* 15)	SG 200 (*n =* 15)
Total intake (Kcal/day)	Baseline	1923.50 ± 345.20	2117.10 ± 256.7 20	2227.80 ± 234.43
	Final	2234.10 ± 421.60	2005.10 ± 341.60	1927.40 ± 342.10
Proteins (g/day)	Baseline	125.30 ± 25.80	130.20 ± 17.20	127.10 ± 23.12
	Final	128.40 ± 27.60	127.20 ± 19.80	133.50 ± 34.10
Carbohydrates (mg/dL)	Baseline	283.00 ± 63.10	255.70 ± 32.00	251.34 ± 65.70
	Final	288.10 ± 56.20	253.60 ± 68.20	268.21 ± 43.20
SFA (g/day)	Baseline	24.21 ± 20.13	21.03 ± 19.70	22.35 ± 16.40
	Final	28.45 ± 26.40	20.70 ± 20.30	24.12 ± 19.20
MFA (g/day)	Baseline	27.89 ± 120.00	29.34 ± 34.00	28.56 ± 21.30
	Final	28.34 ± 21.30	27.67 ± 35.10	30.31 ± 45.10
PUFA (g/day)	Baseline	11.78 ± 4.12	12.54 ± 3.45	12.03 ± 5.60
	Final	11.02 ± 5.60	13.21 ± 2.56	11.40 ± 2.34
Cholesterol (mg/day)	Baseline	328.10 ± 87.10	340.23 ± 89.45	338.45 ± 65.70
	Final	330.23 ± 78.30	338.67 ± 67.80	343.67 ± 65.90

SG 25: 25 mg group; SG 200: 200 mg group; SFA: saturated fatty acids; MFA: monounsaturated fatty acids; PUFA: polyunsaturated fatty acids; g: grams; Kcal: kilocalories.

**Table 3:** Anthropometric values during the study.

		PG (*n* = 16)	SG 25 (*n* = 15)	SG 200 (*n* = 15)
Total weight (kg)	Baseline	72.14 ± 7.18	71.28 ± 8.26	72.29 ± 11.13
	Month 1	72.18 ± 6.86	72.00 ± 8.55	72.00 ± 11.40
	Month 2	72.86 ± 6.90 ^Ω #^	71.10 ± 7.50	72.00 ± 10.81
	Desadaptation	73.12 ± 7.05 ^Ω µ^	72.60 ± 8.03	72.41 ± 11.12
Ʃ6 skinfolds (mm)	Baseline	85.27 ± 23.99	82.14 ± 20.21	87.11 ± 23.50
	Month 1	86.45 ± 30.24	79.49 ± 21.50	80.95 ± 21.35
	Month 2	88.95 ± 30.00	77.74 ± 24.70	80.31 ± 20.30 ^Ω^
	Desadaptation	88.03 ± 29.36	85.42 ± 31.30 ^#^	89.95 ± 27.07
Fat percentage (%)	Baseline	12.84 ± 2.11	12.98 ± 2.34	13.4 ± 3.65
	Month 1	13.48 ± 3.12	12.10 ± 2.37	12.94 ± 2.81
	Month 2	13.94 ± 2.02	11.08 ± 2.93 ^Ω^	11.10 ± 2.85 ^Ω^
	Desadaptation	13.97 ± 2.55	11.93 ± 3.04	12.17 ± 3.66
Muscle percentage (%)	Baseline	47.82 ± 2.75	47.39 ± 2.50	47.12 ± 2.56
	Month 1	47.11 ± 2.50	48.47 ± 3.07	48.79 ± 4.77
	Month 2	47.89 ± 2.81	48.91 ± 2.10 ^Ω^	48.28 ± 2.10 ^Ω^
	Desadaptation	47.17 ± 2.27	48.13 ± 3.10	48.66 ± 5.16

SG 25: 25 mg; SG 200: 200 mg; ^#^ *p* < 0.05 differences with respect to previous evaluation (paired samples Wilcoxon test); ^µ^ *p* < 0.05 differences mismatch vs. month 1 (paired samples Wilcoxon test); ^Ω^ *p* < 0.05 differences mismatch or month 2 vs. baseline (paired samples Wilcoxon test).

**Table 4:** Hormonal values at the evaluations.

		PG (*n* = 16)	SG 25 (*n* = 15)	SG 200 (*n* = 15)
Erythropoietin (IU/L)	Baseline	10.18 ± 1.60	10.00 ± 3.33	9.49 ± 1.76
	Month 1	9.93 ± 2.03	10.08 ± 3.12	9.39 ± 2.72
	Month 2	10.05 ± 2.06	11.63 ± 3.35 ^Ω^	10.76 ± 2.75 ^Ω^
	Desadaptation	9.64 ± 2.37	9.86 ± 4.09 ^#^	10.25 ± 3.28
Free testosterone (pg/mL)	Baseline	21.39 ± 5.04	18.31 ± 4.03	22.4 ± 5.28
	Month 1	18.51 ± 5.51	22.67 ± 11.45	24.18 ± 7.58 *
	Month 2	18.98 ± 5.67	21.55 ± 5.63 ^Ω^	23.73 ± 9.99
	Desadaptation	22.84 ± 8.87 ^µ^	21.87 ± 6.13 ^Ω^	26.71 ± 11.80
Cortisol (mcg/dL)	Baseline	12.51 ± 2.81	12.55 ± 2.30	14.02 ± 3.20
	Month 1	12.78 ± 2.60	12.67 ± 2.69	12.5 ± 2.84
	Month 2	13.62 ± 3.09	12.57 ± 3.43	13.07 ± 3.44
	Desadaptation	14.13 ± 2.83 ^Ω µ^	15.01 ± 3.53	15.04 ± 2.74
DHEA (mcg/mL)	Baseline	3.43 ± 0.90	3.30 ± 0.91	4.16 ± 1.35
	Month 1	3.50 ± 1.22	3.43 ± 1.17	4.16 ± 1.23
	Month 2	3.33 ± 1.18	3.41 ± 1.06	4.20 ± 1.03 *
	Desadaptation	3.97 ± 1.94 ^#^	3.91 ± 1.33 ^Ω^	4.32 ± 1.09
GH (ng/mL)	Baseline	0.83 ± 1.93	0.32 ± 0.41	0.51 ± 0.94
	Month 1	0.60 ± 1.13	0.64 ± 1.08	0.45 ± 0.60
	Month 2	0.34 ± 1.01	1.12 ± 1.90	0.26 ± 0.43
	Desadaptation	0.14 ± 0.17 ^µ Ω^	0.12 ± 0.12	0.11 ± 0.12 ^µ^
IGF-1 (ng/mL)	Baseline	269.68 ± 65.55	253.25 ± 60.9	260.5 ± 70.92
	Month 1	278.81 ± 77.71	298 ± 129.68	314.67 ± 140.53
	Month 2	276.59 ± 63.90	361.58 ± 129.77 ^Ω Ω #^	382.66 ± 147.67 ^Ω Ω #^
	Desadaptation	271.87 ± 58.97	420.31 ± 140.45 ^Ω Ω µ^	379.08 ± 91.61 ^Ω Ω^

SG 25: 25 mg group; SG 200: 200 mg group; GH: growth hormone; DHEA: dehydroepiandrosterone; IGF-1: insulin-like growth factor 1; ^#^ *p* < 0.05 differences with respect to previous evaluation (paired samples Wilcoxon test); ^µ^ *p* < 0.05 differences desadaptation vs. month 1 (paired samples Wilcoxon test); ^Ω^ *p* < 0.05 desadaptation or month 2 vs. baseline; ^Ω Ω^ *p* < 0.01 desadaptation or month 2 vs. baseline (paired samples Wilcoxon test; * *p* < 0.05 differences placebo vs. SG 200 or SG 25 (Mann–Whitney U test).

**Table 5:** White blood cell and platelet counts at the evaluations.

		PG (*n* = 16)	SG 25 (*n =* 15)	SG 200 (*n =* 15)
Leukocytes (Thousands)	Baseline	5.89 ± 1.02	5.85 ± 1.22	6.64 ± 2.29
	Month 1	5.66 ± 1.28	6.28 ± 1.13 ^#^	6.16 ± 1.45
	Month 2	6.39 ± 1.33 ^#^	6.27 ± 1.19	5.97 ± 1.15
	Desadaptation	6.37 ± 1.42 ^µ^	7.25 ± 1.88 ^Ω^	6.5 ± 1.16
Neutrophils (10^3^/µL)	Baseline	3.64 ± 0.70	3.4 ± 0.68	4.45 ± 1.88
	Month 1	3.37 ± 0.80	3.54 ± 0.65	3.7 ± 1.18
	Month 2	4.13 ± 1.19 ^#^	3.55 ± 0.71	3.62 ± 0.82
	Desadaptation	4.06 ± 1.05 ^µ^	4.65 ± 1.69 ^Ω µ^	4.05 ± 0.80
Basophils (10^3^/µL)	Baseline	0.07 ± 0.03	0.08 ± 0.06	0.12 ± 0.08
	Month 1	0.05 ± 0.05	0.14 ± 0.16 ^*^	0.12 ± 0.11
	Month 2	0.07 ± 0.05	0.08 ± 0.06	0.04 ± 0.03
	Desadaptation	0.06 ± 0.06	0.07 ± 0.03	0.07 ± 0.04
Eosinophils (10^3^/µL)	Baseline	0.3 ± 0.24	0.25 ± 0.16	0.25 ± 0.1
	Month 1	0.21 ± 0.13	0.22 ± 0.18	0.22 ± 0.08
	Month 2	0.20 ± 0.11	0.18 ± 0.09 ^Ω^	0.22 ± 0.07
	Desadaptation	0.21 ± 0.12	0.27 ± 0.16 ^#^	0.26 ± 0.1
Monocytes (10^3^/µL)	Baseline	0.47 ± 0.12	0.47 ± 0.15	0.6 ± 0.24
	Month 1	0.42 ± 0.14	0.56 ± 0.21^*^	0.54 ± 0.20
	Month 2	0.50 ± 0.16	0.55 ± 0.22	0.52 ± 0.13
	Desadaptation	0.55 ± 0.15	0.6 ± 0.19	0.6 ± 0.21
Lymphocytes (10^3^/µL)	Baseline	1.42 ± 0.59	1.67 ± 0.47 ^^^	1.26 ± 0.50
	Month 1	1.63 ± 0.56	1.82 ± 0.43	1.58 ± 0.35 ^#^
	Month 2	1.50 ± 0.53	1.92 ± 0.45 ^Ω^ *	1.51 ± 0.57 ^Ω^
	Desadaptation	1.62 ± 0.53	1.67 ± 0.42 ^# µ^	1.52 ± 0.68 ^Ω^
Platelets (thousands)	Baseline	211.81 ± 48.01	268.58 ± 40.12 *	232.08 ± 57.57
	Month 1	197.37 ± 33.98 ^#^	221.50 ± 26.11 ^#^	212.50 ± 32.90
	Month 2	196.00 ± 20.16	222.33 ± 21.03 ^Ω Ω^ *	212.17 ± 38.94
	Desadaptation	190.94 ± 26.56 ^Ω^	217.42 ± 33.90 ^Ω Ω^ *	204.25 ± 34.39
Plateletocrit (%)	Baseline	0.20 ± 0.04	0.25 ± 0.05 *	0.20 ± 0.04 ^^^
	Month 1	0.19 ± 0.03	0.21 ± 0.03 ^#^	0.18 ± 0.04
	Month 2	0.20 ± 0.00	0.20 ± 0.00	0.20 ± 0.04
	Desadaptation	0.19 ± 0.02	0.18 ± 0.04 ^Ω^	0.19 ± 0.03
MPV (fL)	Baseline	8.88 ± 0.93	8.81 ± 0.47	8.49 ± 0.46
	Month 1	8.98 ± 0.96	8.75 ± 0.57	8.53 ± 0.65
	Month 2	8.88 ± 0.91	8.76 ± 0.60	8.52 ± 0.61
	Desadaptation	8.88 ± 0.84	8.67 ± 0.69 ^µ^	8.50 ± 0.67

SG 25: 25 mg group; SG 200: 200 mg group; MPV: mean platelet volume; ^#^ *p* < 0.05 differences with respect to previous evaluation (paired samples Wilcoxon test); ^µ^ *p* < 0.05 differences desadaptation vs. month 1 (paired samples Wilcoxon test); ^Ω^ *p* < 0.05 desadaptation or month 2 vs. baseline (paired samples Wilcoxon test); ^Ω Ω^ *p* < 0.01 mismatch or month 2 vs. baseline (paired samples Wilcoxon test); * *p* < 0.05 differences placebo vs. SG 25 or SG 200 (Mann–Whitney U test); ^^^ *p* < 0.05 differences SG 25 vs. SG 200 (Mann–Whitney U test).

**Table 6:** Red blood cells at the evaluations.

		PG (*n* = 16)	SG 25 (*n =* 15)	SG 200 (*n =* 15)
Red blood cells (millions)	Baseline	5.22 ± 0.64	5.21 ± 0.55	5.05 ± 0.45
	Month 1	5.06 ± 0.29	5.24 ± 0.54	5.11 ± 0.27
	Month 2	5.07 ± 0.21	5.29 ± 0.80	5.15 ± 0.32
	Desadaptation	5.06 ± 0.22	5.19 ± 0.6	5.06 ± 0.26
Hemoglobin (gr %)	Baseline	15.68 ± 1.73	15.05 ± 1.31	15.14 ± 1.37
	Month 1	15.07 ± 0.73	14.95 ± 1.71	14.97 ± 0.67
	Month 2	15.2 ± 0.73	14.98 ± 1.52	15.05 ± 0.57
	Desadaptation	15.2 ± 0.73	15.01 ± 1.52	15.14 ± 0.67
Hematocrit (%)	Baseline	46.31 ± 5.48	45.07 ± 3.51	45.26 ± 4.25
	Month 1	45.31 ± 3.43	45.59 ± 4.27	45.84 ± 2.34
	Month 2	45.47 ± 2.71	45.79 ± 3.34	46.22 ± 2.33
	Desadaptation	44.63 ± 3.15	44.16 ± 3.23 ^µ #^	44.39 ± 2.64 ^µ #^
MCV (fL)	Baseline	87.07 ± 4.05	85.42 ± 9.02	87.93 ± 3.83
	Month 1	87.68 ± 4.11 ^#^	85.77 ± 9.01	87.93 ± 4.30
	Month 2	87.84 ± 4.16 ^Ω^	85.68 ± 9.28	87.73 ± 4.37
	Desadaptation	86.65 ± 4.01 ^#^	84.58 ± 9.42	86.09 ± 4.15 ^µ^
MCH (Pg)	Baseline	29.52 ± 1.49	28.61 ± 3.75	29.41 ± 1.15
	Month 1	29.19 ± 1.27 ^#^	28.13 ± 3.56 ^#^	28.73 ± 1.6 ^#^
	Month 2	29.36 ± 1.25	28.02 ± 3.51 ^Ω^	28.58 ± 1.27 ^Ω^
	Desadaptation	29.50 ± 1.54	28.8 ± 3.45 ^µ #^	29.39 ± 1.34 ^µ #^

SG 25: 25 mg group; SG 200: 200 mg group; MCV: medium corpuscular volume; MCH: mean corpuscular hemoglobin; ^#^ *p* < 0.05 differences with respect to previous evaluation (paired samples Wilcoxon test); ^µ^ *p* < 0.05 differences mismatch vs. month 1 (paired samples Wilcoxon test); ^Ω^ *p* < 0.05 differences mismatch or month 2 vs. baseline (paired samples Wilcoxon test).

## Data Availability

Not applicable.
